# Giant cell tumor of bone and secondary osteoarthritis

**DOI:** 10.1016/j.heliyon.2024.e30890

**Published:** 2024-05-09

**Authors:** Niket Todi, David M. Hiltzik, Drew D. Moore

**Affiliations:** a Corewell Health William Beaumont University Hospital, Department of Orthopaedic Surgery, 3601 W 13 Mile Rd, Royal Oak, MI, 48073, USA; b Northwestern University, Department of Orthopaedic Surgery, 303 E Superior St, Chicago, IL, 60611, USA; c Oakland University William Beaumont School of Medicine, Department of Orthopaedic Surgery, 586 Pioneer Dr, Rochester, MI, 48309, USA

**Keywords:** Giant cell tumor of bone, Secondary osteoarthritis, Musculoskeletal oncology, Total knee arthroplasty

## Abstract

Giant cell tumor of bone is a commonly encountered aggressive epiphyseal bone tumor, most often treated surgically. The natural history and presentation are classically described but the histopathology is poorly understood. Intralesional curettage is the mainstay of treatment, but there is significant variation in the use of adjuvant and cavity filling modalities. No gold standard has been agreed upon for treatment, and a variety of techniques are currently in use. Given its location, secondary osteoarthritis is a known long-term complication. This review examines the natural history of giant cell tumors, treatment options and complications, and subsequent development of osteoarthritis. Arthroplasty is usually indicated for secondary osteoarthritis although data is limited on its efficacy. Further directions will likely center on improved pharmacological treatments as well as improved arthroplasty techniques.

## Highlights

• GCT may represent a neoplastic or reactive process.
• Secondary osteoarthritis is common in patients with history of reoperation.
• Secondary osteoarthritis may be less common with some surgical techniques.

## 1 Introduction

Giant Cell Tumor of Bone (GCT) is a locally aggressive benign neoplasm characterized by multinucleated osteoclast-type giant cells [1]. It is most often found in the epiphysis of long bones. The lesion most commonly causes local tissue destruction but can metastasize to lungs and lymph nodes, and rarely undergo malignant transformation. Definitive treatment usually incorporates surgical intervention, but frequent involvement of the epiphysis and subchondral bone has been associated with a relatively high incidence of secondary osteoarthritis. This complication has been investigated in the literature, however emerging techniques as well as the prolonged time-course of the development of secondary osteoarthritis necessitates careful appraisal of up-to-date research. The goal of this review is to examine recent literature regarding GCT as well as the incidence and risk factors of secondary osteoarthritis following surgical resection of epiphyseal GCT.

## 2 Epidemiology

GCTs represent 3–5% of primary bone tumors in the United States and Europe, and 15–21 % of benign bone tumors [2]. The incidence has been estimated at 1.7 per million people and is reportedly higher in Asian populations. In China, they have been reported to represent 20 % of primary bone tumors [3], and it has been estimated that 18 % of all non-hematogenous primary bone tumors are GCTs in individuals of Asian descent [4]. However, other papers conflict this difference in incidence, with one study finding that the incidence of GCT in the United States is higher than the incidence in China [3]. The Swedish Cancer Registry found GCT in 11 % of bone tumors, with an incidence of 1.3 per million persons per year [5]. A female predominance has been described, with female-to-male ratio ranging from 54 % [2,5] to 60 % [6].

GCT arises most commonly in the third decade of life with 84 % occurring in patients older than 19 [3,7]. Incidence of GCT in skeletally immature individuals has been reported to be between 1.8 and 10.6 % [8]. In skeletally immature individuals, the open physis does not appear to prevent tumor penetration of epiphyseal cartilage [8]. In older individuals, GCTs tend to share histologic and pathologic presentation to younger adults [9]. In general, there are no identifiable risk factors for giant cell tumors. Those with Paget's disease of bone can be at increased risk of developing giant cell tumors, although these studies are confounded by possible genetic clustering [10].

## 3 Clinical presentation

The most common presenting symptom of GCT is pain. Other findings on presentation include soft tissue swelling and deformity as well as pathologic fracture. In one meta-analysis, pathologic fracture was found to occur in 10–35 % (18 % over the total population included in the meta-analysis). Those with pathologic fracture at presentation are noted to have more aggressive disease and higher rate of complications including arthrofibrosis. However, this same study concluded that pathologic fracture did not affect the rate of local recurrence [11]. Pathologic fractures are more common in older individuals with GCTs, with one study showing a rate of 44 % in those over 55 [9].

GCT is most often found in the epiphysis of long bones and can extend into the metaphysis [12]. Only 1.2 % are found outside of the metaphysis. The most common location for GCT is the knee, as 50 % of GCTs are located either in the proximal tibia or distal femur. The next most common locations are distal radius (10 %), sacrum (7 %) [3], and proximal humerus (4 %) [13]. Of note, Chinese epidemiology studies seem to show higher rates of radius involvement than American databases. GCTs are usually solitary lesions; fewer than 1 % are multicentric [14]. Multicentric GCTs tend to be more clinically aggressive with higher propensity for small bones and younger patients. Younger patients have also been reported to have higher rates of vertebral primary tumors in small studies [15], but this has not been observed in analysis of larger sample sizes [8]. Small bone GCT have been noted to be 3–5% of GCTs worldwide and tend to be more locally aggressive [16].

## 4 Imaging

On radiographs, GCTs appear lucent and eccentric in bone. The lesions have an aggressive appearance with local bony destruction, cortical breakthrough, and soft tissue expansion. GCTs lack the dense peripheral sclerosis noted in non-ossifying fibromas (NOFs) and are usually not mineralized. However, calcifications can be seen in metastatic soft tissue GCTs. On MRI, GCTs are well-circumscribed with homogeneous signal intensity. They demonstrate low signal intensity on T1 and intermediate signal intensity on T2. On CT imaging, GCTs demonstrate absence of bone or mineralization within the lesion. Chest CT is the recommended imaging modality to evaluate for lung metastases, although some advocate that chest radiographs are sufficient. Bone scan is often helpful in multicentric disease but limited by specificity. On PET scan, GCTs show high uptake as osteoclasts are metabolically active [17], thus PET scan may be useful in assessing tumor metabolism and angiogenic activity.

## 5 Classification and staging

The Campanacci classification describes the following grades: Grade 1 (latent) consists of well-defined margin and intact cortex; grade 2 (active) consists of well-defined margin but no radiopaque rim, and cortex is thinned and moderately expanded (presence of fracture confers grade IIb); grade 3 (aggressive) consists of indistinct borders and cortical destructions. This classification has no correlation with local recurrence or metastases [18].

The Jaffe histologic grading system was developed in 1940 and describes the following grades: Grade 1 is defined by numerous giant cells without mononuclear cells or mitotic activity; grade 2 consists of mononuclear stromal cells with moderate atypia and mitotic activity; grade 3 consists of giant cells that are few and small, with a greater degree of atypia and pleomorphism with mitotic activity. The Jaffe histologic grading system, as well as other grading systems, have been shown to have little correlation with prognosis [7,19].

## 6 Natural history

Although uncommon, metastasis of GCT can occur and usually presents as a cluster of GCTs within the lung [20]. One study estimated 3 % of GCTs metastasizing to the lungs. Metastasis appears an average of 3–5 years after initial diagnosis of the primary lesion and can be late stage on presentation [21]. Rare non-pulmonary sites have been noted in various case reports. Pulmonary metastases are unpredictable; the lesions can spontaneously regress, remain stable, grow slowly, or rapidly progress [20]. Risk factors for metastases include local recurrence as well as specific cytokine expression. Treatment includes observation, metastasectomy and adjuvant chemotherapy. Pulmonary metastasis has been cited as a cause of death due to GCT, however this is rare, and prognosis is generally good [22].

## 7 Diagnosis

Differential diagnosis of GCT can include brown tumors, aneurysmal bone cysts (ABC), chondroblastoma, osteoblastoma, or osteosarcoma. Metastasis from a vascular tumor is also possible. In the sacrum, giant cell tumors and chordoma can present similarly on radiographs. Due to the wide differential and variability in presentation, biopsy is needed for diagnosis of GCT. Mutations in H3F3A are identified in 96 % of cases arising in long bones, although this mutation is also present in ABCs, NOFs, and chondroblastomas [23]. This mutation appears to be an essential feature of GCTs.

## 8 Histopathology and molecular findings

On gross inspection, GCTs appear chocolate brown or yellow and are soft, spongy, and friable. The yellow to orange coloring is due to hemosiderin, and cystic cavities are common. If the lesion was resected rather than curreted, there will be variable cortical expansion and disruption on pathologic examination. Periosteum is usually intact [24]. ABCs can be found in 14 % of GCTs, and this is suspected to be due to local hemorrhage or reactive hyperplasia of bone and aneurysm formation [25]. Secondary ABCs can be a risk factor for recurrence of GCTs. However, the histopathology of ABCs is unclear and overall, it is not known whether they are truly secondary lesions with GCTs [26].

On histology, lesions are cellular with characteristic multinucleated giant cells in a background network of stromal mononuclear cells. The mononuclear stromal cells are plump, round, oval, or spindle shaped. The background stroma displays prominent mitotic activity without atypia, and mitotic activity does not affect grading of the lesion. Multinucleated giant cells have centrally located nuclei as opposed to Langerhans-type giant cells seen in atypical infections. Nuclei are compact, similar in appearance to those seen in stromal cells, and thus giant cells may represent a syncytium of stromal cells. Multinucleated giant cells can exceed 50 % of the total cell content of the sample and are likely derived from circulating monocytes [27]. They are non-neoplastic. Tumors were found to have vascular permeation in 5 % of cases, but this did not correlate with prognosis [19].

Committed monocytoid pre-osteoclasts positive for receptor activator for nuclear factor κB (RANK) are represented in the marrow and circulate in the periphery. These cells can respond quickly to RANK ligand (RANKL) and represent a potential marker for GCT detection. Giant cells also express tartrate resistant acid phosphatase (TRAP) and vitronectin receptor (VNR). Mononuclear cells express alkaline phosphatase, RANKL, osteoprotegerin (OPG), and TNF-related apoptosis-inducing ligand (TRAIL). Lacunar resorption by multinucleated giant cells is inhibited by OPG, zoledronate, and calcitonin [28]. GCT tissue has also been found to contain stromal cell derived factor 1 (SDF-1), a chemoattractant for hematopoietic osteoclast precursor cells, in concentrations sufficient for recruitment [29]. Connexin43 downregulation has also been shown with reduced progression free survival and worse outcomes [30].

The histogenesis of GCT is incompletely understood; it may be neoplastic or reactive. Supporting evidence in favor of a neoplastic histogenesis include findings of telomeric fusions, increased telomerase activity, and karyotypic aberrations. Amplifications of 20q11 are found in 54 % of GCTs, and overexpression of p53 has been documented and is associated with recurrence. [26]. However, the true neoplastic component of GCT has proven difficult to elucidate; one widely accepted model is that neoplastic osteoblast-like stromal cells secrete RANKL, which stimulates recruitment of osteoclastic cells. However, this is not supported by investigations which found that RANKL is highly expressed in the multinucleated giant cell component of the tumor rather than the stroma, and no deletions or amplifications were found in the stromal cells [24]. This lack of stromal cell cytologic features of malignancy indicates that GCT may be primarily a reactive process [26].

P63 has been noted to be expressed in mononuclear cells of GCT. It is not expressed in other tumors that can have giant cells as part of their histology such as aneurysmal bone cysts and chondroblastoma [16]. Histochemical analysis identifies P63 expression in 96.8 % of giant cell tumors of bone but strong staining is only present in 48.4 % of cells [31]. However, a positive stain is not specific for GCTs and can be seen in NOFs and giant cell rich osteosarcoma. P63 expression has also been associated with higher recurrence rates and could be a prognostic biomarker [32].

## 9 Nonsurgical treatment

Nonsurgical treatments for GCT include radiation, ablation, embolization, denosumab, and bisphosphonates. Radiation, ablation and embolization therapy is recommended only when surgical treatment is not feasible [33]. Surgical treatment is contraindicated if the lesion is inaccessible or if there is significant morbidity for reoperation risk [34]. Radiation dose recommendations vary, ranging from 25 to 70 Gy [35]. Radiation therapy in difficult tumors has been shown to achieve local control in 85 % of cases with a recurrence rate of 10–15 % [36]. However, there has long been a known association with radiation therapy and malignant transformation of GCT [37]. These studies are largely from the era of orthovoltage radiation. Additionally, recent studies have called into question the strength of this correlation when examining megavoltage radiation, estimating the rate of malignancy following radiation therapy at 4.8 % [38]. Malignant transformation of GCTs is confounded by many factors; for example, some cases of malignant tumors following GCT radiotherapy may represent radiation associated sarcoma [37].

One pharmacological option for unresectable GCT in adults is denosumab, a monoclonal antibody against RANKL [39]. Approval of denosumab was based on two trials involving 305 patients with recurrent, unresectable, or high-risk surgery. Malignant transformation has been noted in patients who had GCT treated with denosumab [40]. Eighteen cases of malignant transformation have been noted during denosumab treatment [41]. Recurrence rates of GCT after surgery with denosumab as an adjuvant or neoadjuvant are currently being investigated. Local recurrence rates have been shown to increase when denosumab therapy is combined with surgery, with one study reporting 60 % recurrence with curettage and neoadjuvant denosumab compared to 16 % with curettage alone [[42], [43], [44]]. It is speculated that denosumab treatment results in thickened tumor margin wall as stromal cells are not targeted, hence allowing tumor cells to persist at the margins [41]. Thus, there is concern that recurrence can occur once denosumab treatment ends. However, there is also evidence that neoadjuvant therapy with denosumab can reduce tumor recurrence following intralesional curettage [45]. A randomized clinical trial to confirm this was recently performed which has found no significant differences in the 2-year follow-up period, although the study experienced poor patient accrual and was performed on only 18 patients total. Notably, the authors made specific mention of the notion that reactive bone surrounding the tumor could harbor cells that contribute to recurrence, and reported that they removed this thickened margin using a high-speed burr as part of the study protocol [46].

## 10 Surgical treatment

Surgical management is the mainstay of treatment for GCT. This includes a variety of options including curettage with or without bone graft or polymethyl methacrylate bone cement (PMMA), as well as resection with or without adjuvant therapy. Intralesional curettage in general is the treatment of choice, with the notable exception of GCT located in expendable bones such as the proximal or distal fibula and the distal ulna, which are often simply resected en bloc. Curettage results in good functional and oncologic outcomes but is associated with a recurrence rate of 18–55 % [47,48].

Long bone resection usually requires allograft and either arthrodesis or endoprosthesis, such as proximal femoral replacement [49]. In areas such as the proximal femur, intralesional curettage may not provide enough stability to withstand large shear forces, and arthroplasty options may be indicated [50]. Recurrence rates following resection vary depending on the location of the lesion. One study comparing curettage versus resection and allograft in proximal humerus GCT noted 7 % recurrence in the curettage group and 15 % in the resection group. Patients in the segmental resection group also experienced worse outcomes [51]. Elsewhere, performing a resection and arthrodesis is associated with lower recurrence rates than intralesional treatment, while one study of distal radius GCT demonstrated that functional outcomes between the two approaches are no different. Patients who did have recurrence after intralesional treatment underwent resection and arthrodesis and these secondary procedures had no difference in outcomes. Thus, curettage remains a viable option for initial treatment [52]. Even in the case of pathologic fractures, while recurrence is higher for intralesional treatment, complication rate is higher for reconstruction [53]. Furthermore, in tumors classified as Campanacci 2 with pathologic fracture, intralesional treatment has been shown to have similar recurrence rates compared to en bloc resection [54]. In cases of recurrence, repeat curettage has a cure rate of 80–90 %, supporting curettage over resection [13]. Resection is usually recommended with extensive soft tissue extension or fracture through recurrence or lack of structural integrity.

Other options for reconstruction include distraction osteogenesis with the use of an external fixator, especially in a younger patient [55]. Tsuchiya et al. [56] have shown the use of Ilizarov technique in managing large subarticular defects with good functional outcomes. Osteoarticular allografts can be used in the distal radius and have good functional outcomes with low rates of recurrence [57]. Osteoarticular allografts have been shown to have similar functional outcomes to wrist arthrodesis [58]. Large defects around the knee can be filled with vascularized fibular autograft with good functional outcomes [59].

PMMA is often chosen over bone graft to replace lost bone, as the exothermic reaction of the curing process can cause thermal necrosis of tumor cells that remain in the cavity following curettage [60]. Use of PMMA when compared to autograft is generally a lower cost, is a more readily available option, not associated with donor site morbidity and shown to improve immediate structural stability. Systematic reviews have shown that use of PMMA is associated with lower recurrence rates than bone grafting when reconstructing giant cell cavities [61]. Barium sulfate in PMMA is radiopaque, which allows for detection of tumor recurrence [22]. Kafchitsas et al. [62] demonstrated that an increase of the radiolucent zone was seen in 80 % of patients with recurrence. Drawbacks of PMMA include difficulty of removal in reoperation and revision procedures, and more theoretical risk to patients [63].

One additional technique for intralesional treatment of GCT is the use of a burring tool to thoroughly remove the tumor. Friction from the burr, like the chemical reaction responsible for curing of PMMA, adds a thermal component to the eradication of the tumor. Supplementing curettage with burring has been shown to reduce recurrence by 12–25 % and reduce the need for adjuvant therapy, as these patients have been shown to receive no additional benefit from adjuvants [64].

Adjuvant therapy though useful, provides limited benefit, and successful treatment depends more on thoroughness of intralesional curettage than the specific adjuvant used [65]. Phenol is a toxic compound introduced after curettage that penetrates tissue to a depth of 1–2 mm resulting in coagulation of cytoplasm and cell death. As it is systematically toxic, high morbidity occurs if surrounding tissues are exposed, and the compound can be absorbed through cancellous bone. Use of phenol is associated with a tumor recurrence rate of 5–17 % [66]. Ethanol has been shown to have similar recurrence rates of phenol without the risk of chemical burns [67].

Cryotherapy with liquid nitrogen is another adjuvant to surgical management. Caveats to the use of liquid nitrogen are that it must be allowed to evaporate, and the area must be irrigated to prevent thermal injury. Notably, fracture is a known complication occurring in up to 25 % of cases, therefore prophylactic fixation is sometimes performed [65]. Recurrence rate of GCT following adjuvant therapy with liquid nitrogen is 2–12 % [68].

Adjuvant therapy using an argon beam is considered effective for tissue dessication and coagulation to a depth of 2–3 mm, and this technique avoids the systemic complications and necrosis associated with phenol and cryotherapy. Comparison with phenol demonstrates similar recurrence rates while avoiding the toxicity of phenol [69]. Use of the argon beam is associated with a recurrence rate of 7 %, but there is currently a scarcity of long-term data [70]. Microwave therapy has also been shown to be effective in preventing recurrence [71].

Bisphosphonates as adjuvant therapy are generally considered safe and mixing the medication with PMMA can reduce systemic side effects. Local use of bisphosphonates is not associated with systemic complications such as avascular necrosis of the jaw or atypical subtrochanteric fractures [72]. Treatment of GCT with curettage and implantation of bisphosphonate-loaded bone cement is associated with a recurrence rate of 5.9 %. However, an improvement in outcomes has not been clearly elucidated [72]. Calcitonin has also been used as adjuvant but has not been shown to be effective [73].

It is likely that overall adequacy of tumor removal determines the risk of recurrence rather than which adjuvant modality is used [65]. In general, recurrence rates after curettage procedures are variable, ranging from 27 to 65 % for bone grafting, and 12–42 % for PMMA [74]. Adjuvant therapies seem to decrease the recurrence rate to 12–27 % in the modern era [75]. Less than 5 mm of residual thickness of subchondral bone tends to be associated with recurrence [76]. Conversely, resection has extremely low rates of recurrence. In general, most tumor recurrence occurs within 3 years [75].

## 11 Metastatic disease and malignant transformation

Metastasis and malignant transformation of GCT is uncommon but well documented. Metastases to the lungs are the most common and are considered benign pulmonary implants. Estimates of rates of lung metastasis vary, ranging from 1 to 9% [77], with one study of 368 cases identifying a rate of 7.5 % [21]. Higher rates of metastasis have been reported from GCT located in the spine [78]. These pulmonary metastases generally have good outcomes and do not change prognosis [79,80]. Some literature cites local recurrence as the only risk factor for metastasis [21], but other documented risk factors include young age, locally aggressive lesions, and axial vs. appendicular lesions [81]. Although lung metastasis is largely considered benign, it has been documented as a cause of death [22]. The treatment of choice for pulmonary metastases is wide surgical resection [20]. Chemotherapy and radiation can be used as adjuvants to surgical resection or as solitary agents if resection is not possible [82]. Interferon alfa-2b has also shown to be successful in the treatment of metastatic or recurrent GCT by inhibiting angiogenesis [83]. However, there is evidence that nodules under the size 5 mm can be observed safely as only half of the metastases progress [84].

Sarcomatous transformation of GCT has been documented, including transformation to osteosarcoma, fibrosarcoma, and malignant histiocytoma [85,86]. Incidence of malignancy is estimated at 4 % [38]. However, it is difficult to determine whether these cases represent true malignant transformation as opposed to spontaneous lesions such as post-radiation sarcoma or misdiagnosis of giant-cell-rich osteosarcoma [85]. Generally malignant GCT is considered as a high-grade sarcoma although prognosis is not well studied.

## 12 Surveillance

Surveillance of malignant transformations of GCT is like that of sarcomas [35]. This includes serial positron emission scans and radiography of the tumor site and of the chest, to monitor for pulmonary metastasis. When GCT recurs, it is often associated with new pain and swelling. Risk factors for recurrence include ABC, incomplete resection at tumor margins, and younger age [25,87,88], although increased recurrence in younger patients has not been consistently demonstrated [88]. It has also been shown that extension of the lesion into soft tissues increases local recurrence by a factor of 4. Size of the primary lesion does not correlate to rate of recurrence [88]. In the event of local recurrence, additional surveillance consists of chest imaging a minimum of 3 years after the diagnosis of local recurrence is made, as an estimated 85 % of metastases with local recurrence occur within that time frame [21].

Delayed treatment can negatively impact outcomes in GCT. Pathologic fracture often results in poorer outcomes and increasing involvement of subchondral bone increases the risk of developing OA. As GCT extends into soft tissue, risk of metastasis is also increased.

## 13 Secondary osteoarthritis

As giant cell tumors are epiphyseal in nature, osteoarthritis of the adjacent joint is a long-term proposed consequence. Most of the research focuses on weight bearing joints but still there is limited data on actual incidence of osteoarthritis in these joints. It has been theorized that subchondral cement may predispose patients to osteoarthritis [7]. Radin et al. [89] demonstrated that repeated load to the knee of the rabbit lead to subchondral fractures and then further knee joint degeneration. Szalay et al. [90] showed that 84 % of GCTs had subchondral involvement. Pathologic fractures are assumed to increase the risk of developing degenerative changes, but current evidence is largely case reports [91]. Septic complications are also assumed to be associated with secondary osteoarthritis. Reoperation has been found to be among the most significant risk factors for development of secondary osteoarthritis [76].

Early studies showed that PMMA was associated with higher rates of articular degeneration than bone grafting [65]. However, this data is mixed at best. The exothermic reaction induced by PMMA can also cause damage to the subchondral bone and possibly the cartilage. Finite element analysis has demonstrated that if less than 3 mm of cancellous bone remains in the subchondral region prior to introduction of PMMA, the region can be exposed to necrotic conditions as the cement cures [92]. In addition, Welch et al. [93] described the development of a sclerotic rim at the bone-cement surface, reducing the mechanical advantage of subchondral bone. Larger involvement of subchondral bone is associated with poor outcomes [94]. Thus, some surgeons believe that PMMA can induce secondary osteoarthritis.

Rate of osteoarthrosis for patients undergoing surgery involving PMMA is estimated at 12 % in short term follow-up in initial studies describing cementing techniques. Other studies have shown degenerative changes in 26–33 % of patients, and one retrospective study with a minimum follow-up of 10 years reported a rate of 21 %, although only 8 % experienced progression of their arthritis during the follow-up period [74,76,95,96]. Some studies have shown that PMMA is an independent risk factor for total joint replacement [63]. However, it has also been shown that subchondral PMMA does not cause joint degeneration [6]. One study found higher rates of osteoarthritis in patients who received bone graft versus PMMA at 24 months, although there was no significant difference at 50 months [90]. Von Steyern et al. [97] showed no evidence of arthritis at 11-year follow-up with cement, apart from one patient who had an intra-articular fracture and reoperation. Similarly, Wada et al. [91] also reported low rates of osteoarthritis with PMMA without bone graft aside from one patient with intra-articular fracture at presentation. Van der Heijden et al. [98] showed that at 7 years median follow-up after GCT curettage with PMMA, 17 % of patients had radiographic evidence of arthritis. However, patients with severe arthritis were not severely symptomatic, indicating limited clinical effect. Similarly, in a retrospective study of 5 patients with greater than 20 years of follow-up after GCT excision with curettage and PMMA, Kito et al. reported that all had good limb function without need for arthroplasty at final follow up despite all having radiographic evidence of joint degeneration [99] (Table 1).

**Table 1:** Reported rates of secondary osteoarthritis following GCT resection and corresponding interventions.

Author	Year	Intervention	n	Mean Follow-up	Rate of OA	Additional Notes
Araki et al. [74]	2020	Calcium phosphate	19	131mo	26 %	
Takeuchi et al. [100]	2018	Calcium phosphate	26	87mo	4 %	
Kito et al. [99]	2018	PMMA alone	5	336mo	40 %	OA defined as Kellgren-Lawrence (KL) grade 3 or greater
van der Heijden et al. [98]	2013	PMMA±Bone graft	53	86mo	17 %	OA defined as KL 3 or greater
von Steyern et al. [97]	2007	PMMA alone	9	132mo	11 %	Mean distance from cement to cartilage: 1 mm
Wada et al. [101]	2002	PMMA alone	15	46mo	7 %	
Wu et al. [102]	2018	Bone graft + PMMA	27	33mo	11 %	All were KL2 or less
Benevenia et al. [103]	2017	Bone graft alone	4	59mo	0 %	
		Bone graft + PMMA	17		6 %	
		PMMA alone	22		32 %	
Xu et al. [95]	2013	Bone graft + PMMA	34	35mo	29 %	Reported on patients with <10 mm remaining subchondral bone
		PMMA alone	23		57 %	
Suzuki et al. [76]	2007	PMMA±Bone graft	12	57mo	25 %	Not significant (p = 0.429)
		Bone graft alone	18		38 %	
Szalay et al. [90]	2006	Bone graft alone	44	24mo	14 %	No significant difference beyond 50 months
		PMMA alone	36		8 %	

Degree of GCT involvement of the subchondral region has been correlated with development of arthritis [76,98], and subchondral bone grafts have been shown to be superior to cement for restoration of normal subchondral anatomy [13]. Wu et al. described their surgical technique of extensive curettage, subchondral grafting of 1 cm, PMMA, followed by plating. After mean follow-up of 33 months, the arthritic rate was 11.1 % [102]. PMMA with bone grafting compared to without grafting has been associated with decreased rates of osteoarthritis at a mean follow-up of 5 years [103]. Subchondral grafting has been shown to decrease the rate of secondary arthritis by 27.1 % when compared to subchondral PMMA (29.4 % vs 56.5 %) in patients who have less than 10 mm of remaining subchondral bone following curettage [95]. Van der Heijden et al. further characterized the influence of subchondral bone involvement and found that the area of subchondral bone involved had greater effect than the thickness of remaining subchondral bone (Hazard Ratio of 9 when subchondral involvement >70 % vs. 4 when thickness <3 mm) [98]. It has been theorized that osteosynthesis to support subchondral bone can help reduce the risk of subsequent osteoarthritis, but it has been not extensively studied [104] (Table 1).

When calcium phosphate has been used instead of PMMA, Araki et al. [74] showed osteoarthritic progression in 26 % of patients and the survival rate of the joint of 83 % at 10 years. Similarly, Takeuchi et al. [100] had 1 patient out of 26 develop osteoarthritis with the use of calcium phosphate and phenol with curettage (Table 1).

When osteoarthritis develops and fails standard conservative management, there are limited treatment modalities that are available. Definitive management is usually with arthroplasty, especially in the distal femur, proximal tibia or proximal femur. Some surgeons advocate removal of cement and placement of bone graft in staging for arthroplasty [98]. Case reports usually describe the use of revision components with grafting or augments to ensure implant stability with good functional outcomes [105]. Other surgeons have been able to successfully use standard total knee arthroplasty, even in the present of cemented cavities [106]. Zylberberg et al. [106] argue that cement allows for the use of primary implants rather than stems or augments. Conti et al. [107] were able to successfully use a patellofemoral arthroplasty with tantalum cones for secondary patellofemoral arthritis. While there are studies that look at outcomes for arthroplasty after en bloc resection, there is limited data on the use of arthroplasty in secondary osteoarthritis after intralesional treatment.

## 14 Future directions

Many molecular markers have been linked to GCTs, but their clinical use is currently limited. RANKL expression is important to GCT, but its specific role is still poorly understood. PMMA substitutes are being investigated that have similar exothermic reaction but have more favorable osteoconductive and osteoinductive properties [108]. As denosumab is further studied and treatment protocols are developed, it will likely become more of a mainstay of treatment. Navigation can be used to help plan precise cuts in arthroplasty in the presence of cement in case of symptomatic secondary osteoarthritis, preventing the need for revision implants.

## 15 Conclusion

Giant cell tumors are locally aggressive benign tumors that occur in characteristic locations and age groups with poorly understood histopathology. Treatment is usually surgical with intralesional curettage with various adjuvant and cavity filling modalities, although pharmacological adjuvants are being studied. Relatively high rates of recurrence are seen with most treatments and osteoarthritis is a known complication, but subchondral grafting and calcium phosphate as a substitute for PMMA have shown promise in reducing the incidence of osteoarthritis secondary to GCT. Further investigation is needed to characterize the benefits of these techniques.

## Declarations

### Funding

This research did not receive any specific grant from funding agencies in the public, commercial, or not-for-profit sectors.

## Ethics approval

Review and/or approval by an ethics committee was not needed for this study because it was performed on publicly available data.

## Consent to participate

Not applicable.

## Consent for publication

Not applicable.

## Availability of data and material

Has data associated with your study been deposited into a publicly available repository? **No data was used for the research described in the article**.

## Code availability

Not applicable.

## CRediT authorship contribution statement

**Niket Todi:** Writing – original draft, Supervision, Investigation, Conceptualization. **David M. Hiltzik:** Writing – original draft, Investigation. **Drew D. Moore:** Writing – review & editing, Conceptualization.

## Declaration of competing interest

The authors declare that they have no known competing financial interests or personal relationships that could have appeared to influence the work reported in this paper.
